# Asymptomatic Bacteriuria and Anti-Microbial Susceptibility Patterns among Women of Reproductive Age. A Cross-Sectional Study in Primary Care, Ghana

**DOI:** 10.3390/medsci6040118

**Published:** 2018-12-17

**Authors:** Prince Afoakwa, Seth Agyei Domfeh, Bright Oppong Afranie, Dorcas Ohui Owusu, Sampson Donkor, Kennedy Kormla Sakyi, Richard Akesse Adom, Godfred Kyeremeh, Bright Afranie Okyere, Emmanuel Acheampong, Beatrice Amoah

**Affiliations:** 1 Department of Medical Laboratory Technology, Faculty of Health and Allied Sciences, Garden City University College, P.O. Box KS 12775, Kenyasi-Kumasi, Ghana; prinyprince2016@gmail.com (P.A.); sadomfeh@gmail.com (S.A.D.); dorcso.owusu@gnail.com (D.O.O.); godfreddii@gmail.com (G.K.); 2 Department of Molecular Medicine, School of Medical Sciences, Kwame Nkrumah University of Science and Technology, PMB, UPO, Kumasi, Ghana; sampsondonkor08@gmail.com (S.D.); emmanuelachea1990@yahoo.com (E.A.); abeatrice111@gmail.com (B.A.); 3 Department of Microbiology, Tetteh Quarshie memorial hospital, Akuapem Mampong-Korforidua, P.O. Box 26, Ghana; kenritason99@yahoo.com (K.K.S.); akwasioppon977@gmail.com (R.A.A.); 4 Department of Theoretical and Applied Biology, Faculty of Biosciences, Kwame Nkrumah University of Science and Technology, PMB, UPO, Kumasi, Ghana; richardasareafranie@gmail.com

**Keywords:** bacteria, culture, urinary tract infections, urinalysis

## Abstract

*Background:* Asymptomatic bacteriuria (ASB) poses serious future clinical repercussions for reproductive women. The study determined the prevalence of asymptomatic bacteriuria along with anti-microbial susceptibility patterns among women of reproductive age in a primary care facility. *Method:* The study recruited a total of 300 women of reproductive age attending the Tetteh Quarshie Memorial Hospital at Akuapem-Mampong, Ghana, between January and March 2018. Questionnaires were administered to obtain demographic data and predisposing risk factors of ASB. An early-morning midstream urine sample was collected from participants. Urinalysis, urine culture, and anti-microbial susceptibility testing were performed. *Results:* The mean age of participants was 25.43 years. The overall prevalence rate of ASB was 40.3%. The prevalence was higher among pregnant women compared to non-pregnant women (33.3% vs. 7.0%). The most common bacterial isolate was *E. coli* (47.0%) followed by *Proteus* spp. (36.4%), *Klebsiella* spp. (8.3%), and *E. faecalis* (8.3%). Leukocyturia (35.0%) followed by nitrate (30.0%) were the most common urine abnormalities identified on dipstick urinalysis. Most bacteria isolates showed increased resistance to ampicillin (95.04%) and tetracycline (95.04%) while most of the bacterial isolates were sensitive to levofloxacin (94.35%). Demographic characteristics including age (*p* < 0.001), educational level (*p* < 0.001), residency (*p* = 0.001), and marital status (*p* = 0.005) were significantly associated with ASB. Lifestyle characteristics such as sexual status (*p* = 0.001) and frequency of washing of intimate parts after sexual intercourse (*p* < 0.001) were also significantly associated with ASB. *Conclusion:* Asymptomatic bacteriuria, particularly *E. coli* and *Proteus* spp. are prevalent in the urine of pregnant women living in Akuapem-Mampong municipality. Hence public education along with early screening of ASB is essential to reducing future risk of reproductive health complications. Future studies are required to assess the impact of public health on the rate of bacterial infections.

## 1. Introduction

Asymptomatic bacteriuria (ASB) is the second principal bacteria associated infections in primary care [1]. It affects millions of people globally, especially in developed countries [2]. It is estimated that about 20% of women develop ASB during their lifetime and many will have recurrent episodes [3]. Higher incidence rate of infection in females has also being link with sexual activity and child-bearing [4]. Moreover, women are mostly at risk for ASB due to the short nature of their urethra, and some social factors that include personal hygiene and sexual activity [3,5].

Asymptomatic bacteriuria is defined as the presence of active bacteria in the urine of an individual without apparent signs or symptoms of a urinary tract infection (UTI). ASB has been shown to be a significant predictor of UTI, with prevalence rates ranging from 5–6% in healthy women 18–40 years of age and up to 20% in the ambulatory elderly [6]. While some studies [7,8,9] have reported the overall prevalence of ASB particularly in pregnancy to be between 10.0% and 40.0%, a few studies have also reported different prevalence rates of ASB ranging from 13.3% to 28.8% [10]. Previous studies [11,12,13] in the Ghanaian population have reported high ASB prevalence rates. A cross-sectional study conducted among pregnant women attending antenatal clinic reported a 7.3% prevalence rate of ASB [11]; another cross-sectional study by Gyansa-Lutterodt et al. reported a prevalence rate of 31.6%, while a recent study found that 9.6% of female tertiary students tested positive for asymptomatic bacteriuria [14]. Despite the high prevalence rates reported in previous studies, most authors have not adequately reported on the anti-microbial susceptibility pattern as well as possible risk factors associated with ASB.

Anti-microbial drugs play a significant role in reducing the morbidity and mortality associated with microbial infection. Uncomplicated UTIs are usually treated with antibiotics, which unfortunately become a challenge as a results of the resistance developed by the causative pathogens [15]. There is the need for substantial evidence that screening for and treating ASB and the use of anti-microbials with less resistance will decrease the incidence of pyelonephritis, a complication of ASB among women.

It is against this background that the study sought to provide baseline evidence for policy makers and government organizations to formulate and develop risk reduction strategies to address these issues. Therefore, the study determined the prevalence of asymptomatic bacteriuria and antibiotic sensitivity among women of reproductive age attending a primary care facility in Ghana.

## 2. Materials and Methods

### 2.1. Study Design/Study Setting

This cross-sectional study was conducted at the Tetteh Quarshie Memorial Hospital at Akuapem-Mampong. Akuapem-Mampong lies on the Akuapem Mountains in the eastern part of Ghana with a population of about 90,000 inhabitants (Census, 2010). The major occupation of the people in the catchment area is trade with a few working as public servants. The 123-bed compliment hospital serves people spanning the entire semi-urban Akuapem Ridge, its rural valley, and beyond. It is the only government hospital within a radius of 30 km, the nearest being the Regional Hospital, Koforidua, some 35 km northeast. The hospital provides curative services in the following units: obstetrics and gynecology, pediatrics, surgery, medicine, ophthalmology, ear nose throat, psychiatry, and dentistry. Consequently, the hospital handles general diseases such as malaria, anemia, respiratory diseases, hernia, fractures, pregnancy and related diseases, and gynecological diseases.

### 2.2. Study Population and Subject Selection

A simple random-sampling technique was used to recruit a total 300 asymptomatic reproductive women aged 18 to 39 years with no clinical signs and symptoms of UTI who were visiting both the outpatient department and antenatal clinic of Tetteh Quarshie Memorial Hospital. Each woman was given a number, and then a table of random numbers was used to decide which patient to include. Depending on the total number of patients per clinic, a range of 20 to 24 patients were randomly sampled weekly until the sample size was achieved.

### 2.3. Ethical Considerations

The study was approved by the Committee on Human Research Publications and Ethics (CHRPE) at the School of Medical Sciences, Kwame Nkrumah University of Science and Technology (KNUST), Kumasi, Ghana, in collaboration with the management of Tetteh Quarshie Memorial Hospital (CHPRE/AP/146/17, 13th March 2017). Participation was voluntary, and written informed consent was obtained from each participant according to the Helsinki Declaration. Respondents were assured that the information gathered was to be used strictly for research and academic purposes only. In addition, respondents were given the freedom to opt out at any time they thought they could not continue with the study.

### 2.4. Questionnaire Administration and Data Collection

Structured questionnaires were used to obtain socio-demographic and other relevant data from participants who consented. Demographic data such as age, marital status, educational status, occupation, and current residency was recorded. Information on their sexual history was also recorded.

### 2.5. Urine Sampling

Freshly voided, clean-catch mid-stream urine samples of about 10–15 mL were collected using a clean, leak-proof, wide-open, sterile sample container. The samples were sent to the microbiology laboratory and processed within 30 min of collection to ensure the maximum recovery of bacteria.

### 2.6. Sample Culturing Method

Urine was inoculated onto the surface of cysteine lactose electrolyte-deficient (CLED) agar and incubated aerobically at 37 °C for 24–48 h. The plates were then examined macroscopically for bacterial growth. Bacteria colonies were then counted and calculated to find the number of organisms per milliliter specimen using the formula;

$$\frac{\mathrm{Number}\;\mathrm{of}\;\mathrm{colony}\;\mathrm{forming}\;\mathrm{unit}\;(\mathrm{CFU}/\mathrm{mL})}{\mathrm{volume}\;\mathrm{Plated}\times \mathrm{Total}\;\mathrm{dilusion}}$$

### 2.7. Standard Biochemical Identification of Bacterial Isolates

Identification of the working isolates was carried out based on the methods described by Cowan et al. (1993). Biochemical tests such as coagulase, catalase, oxidase, indole, sugar test, urease, and the triple sugar iron test were used to identify bacterial colonies.

### 2.8. Chemical Examination of the Urine Samples

Dipstick urinalysis strips (Accu-Tell Ref ABTUM-A33) were used to detect the following parameters in the urine provided by the participants: protein, glucose, nitrate, glucose, ketones, blood, and leukocytes.

### 2.9. Anti-Microbial Susceptibility Testing

The anti-microbial susceptibility test was performed using the Kirby–Bauer disc diffusion test. The isolates were tested against the following antibiotics: ampicillin (10 μg), cefuroxime (30 μg), cotrimoxazole (25 μg), gentamicin (10 μg), tetracycline (30 μg), nalidixic acid (30 μg), nitroforantoin (300 μg) and pipemidic acid (20 μg), cefotaxine (10 μg), ciproflaxacin (5 μg), levofloxacin (5 μg). Zone diameter was measured by Clinical Laboratory Standard Institute (CSLI) [16,17].

### 2.10. Data Analysis

The data collected was entered with Microsoft Excel and analyzed with SPSS version 23 (SPSS Inc., Chicago, IL, USA). The basic characteristics were presented by frequencies, and categorical data and Chi-square analysis were used to find relationships between categorized variables. A regression model with robust error variance was used to estimate prevalence-rate ratios (PRR) as a measure of association for the relationship between independent variables and UTI status. PRR was used as a measure of association because of the prevalence of the outcome (>10%), thus providing a better estimate of risk than the odds ratio (OR) [18,19]. *p*-values of less than 0.05 were considered to indicate statistical significance.

## 3. Results

Table 1 shows the distribution of ASB stratified by socio-demographic characteristics. The mean age (SD) of the study participants was 25.43 (standard deviation (SD) = 6.54), with a higher majority of them within the age group 20–24 years (62.3%). Most of the participants were informally employed (51.0%), married (80.5%), and had an education to the junior high school level (39.5%). More than half of the participants (56.7%) had previously resided in an urban slum setting. There was no statistically significant difference in occupational status prevalence for ASB, however, significant differences were observed in age groups (*p* < 0.001), educational level (*p* < 0.001), residency (*p* = 0.001), and marital status (*p* = 0.005) for ASB. Patients within the age range of 20–24 years were two times more likely to have ASB compared with those below 20 years of age (PRR = 2.4, *p* = 0.002). With respect to where study participants resided, those in the urban slum showed a higher likelihood compared to rural dwellers (PRR = 1.5, *p* = 0.006).

**Figure 1:**
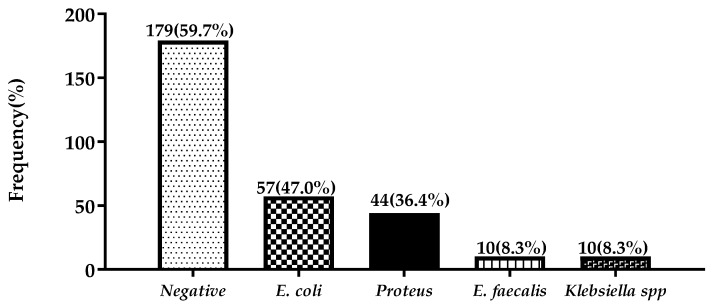
Frequency distribution of bacterial isolates.

Figure 1 shows the frequency distribution of bacterial isolates in urine, the overall prevalence of uropathogens was 40.3% (121/300). The most common bacterial isolate was *E. coli* (47.0%), followed by *Proteus* spp. (36.4%), *Enterococcus fecalis* (8.3%), and *Klebsiella* spp. (8.3%).

Table 2 shows the distribution of ASB stratified by lifestyle characteristics and some other factors. There was a significant difference in ASB prevalence for the department (*p* < 0.001) participants attended at the hospital, sexual relationship (*p* = 0.001), and how frequently participants wash their intimate area after sexual intercourse (*p* < 0.001). Women recruited from the antenatal clinic were more likely to have ASB compared to those who visited the out-patient department (OPD) (PRR = 1.9, *p* < 0.001). Regarding sexual relationships, sexually-active women were less likely to have ASB compared to the non-sexually-active women (PRR = 0.4 (0.1−0.5), *p* < 0.001)).

As shown in Table 3, leukocyturia (35.0%), followed by nitrate (30.0%), proteinuria (8.3%), glucosuria (2.3%), hematuria (1.7%), and ketonuria (1.7%) in that order were the proportion of urine abnormalities observed in the study participants.

Table 4 shows the anti-microbial susceptibility pattern of bacterial isolates. A higher proportion of bacterial isolates were resistant to ampicillin (95.04%) and tetracycline (95.04%) but were highly susceptible to levofloxacin (94.35%) followed by cefotaxine (94.21%), ceftixozime (94.21%), nitrofurantoin (93.87%), and nalidixic acid (91.87%).

## 4. Discussion

The asymptomatic traits of the bacteria that cause urinary tract infection and their anti-microbial patterns make treatment of the disease challenging despite the large number of infections that occur each year. In Ghana, ASB and especially UTI-causing pathogens are considered to be a significant public health burden as they pose serious clinical health outcomes for pregnant women and women of the optimal childbearing age [13]. This study determined the prevalence and risk factors of ASB along with anti-microbial susceptibility patterns among women of reproductive age attending primary care at the Tetteh Quarshie Memorial Hospital in Akuapem-Mampong District.

In this present study, the overall prevalence of ASB was 40.3%. The prevalence was higher in pregnant women (33.3%) compared to non-pregnant women (7.0%). A wide range of prevalence rates have been reported in several cross-sectional studies of pregnant women in the Ghanaian population, Labi et al reported ASB prevalence rate of 5.5% [10], Obirikorang et al. reported a prevalence of 9.5% [11] and Boye et al. reported a prevalence rate of 56.9% [13]. Another cross-sectional study by Boye et al. of female tertiary students in a Ghanaian institution reported 9.6% prevalence for ASB [14], while Darko found a prevalence of 20.0% [20]. The global prevalence as reported by Foxman [3] was 25.0%. These disparities in the rates of prevalence could be explained by the differences in sample size and population type. Compared to the previous studies, our study population included both pregnant women and non-pregnant, however, there is insufficient evidence in the literature on ASB in non-pregnant, premenopausal women as a risk factor for UTIs. Moreover, pregnant women are 20 to 30 times more likely to develop pyelonephritis compared to non-pregnant women of the same reproductive age [20]. This could have accounted for the high prevalence of ASB among pregnant women compared to non-pregnant women in this present study. This buttresses our current finding that pregnant women had 1.9 times increased odds of ASB.

Another finding of this present study was the significant association observed between the age group and ASB status. This finding is consistent with cross-sectional study by Emiru et al. [21] among pregnant women in an Ethiopian population. In their study, higher proportions of the study participants who were infected with ASB were in the age group of 20–29 of reproductive women [21]. Women between aged 20–24 years of age in this study were 2.4 times more likely to have ASB. The probable reason for the current finding is that most young women in their optimal childbearing age are sexually active compared to menopausal women. The desire to engage in sexual activities coupled with the use of contraception for protective sex and exposes them to uropathogens [22]. Results from the study revealed that higher proportions of the participants who were infected with uropathogens were sexually active and washed their private parts after sex. All these together put these participants at risk of ASB. Sex is a common cause of ASB in women as it increases the chances of bacterial contamination of the female urethra [3]. The incidence of infection in females increases directly with sexual activity and child-bearing [22]. In addition, a significant association between how frequently the participants wash their intimate part after sex and the prevalence of UTIs was observed, and this is closely related to Foxman’s report [3].

We found that participants who were residents of an urban slum were 1.5 times more likely to have ASB. This finding concurs with reports from a cross-sectional study by Hossan et al. in Bangladesh [23]. Macroscopic examination of urine provides semi-quantitative information about the presence and absence of uropathogens [24]. Particularly, in this present study urine abnormalities such as leukocyturia and nitrates were prevalent among participants (Table 4). This finding is similar to a study carried out by Simerville et al. in an American population [25].

The most commonly isolated bacterial was *E. coli*, followed by *Proteus* spp., *Klebsiella* spp., and *Enterococcus fecalis* (Figure 1). Similar trends have been reported in studies conducted by Ojo and Anibijuwon among women of reproductive age in Nigeria [26] and pregnant women in Ghana [10,11,13,27]. The higher prevalence of *E. coli* could be due to fecal contamination, the preference of organisms from toilets and the shortness of the female urethra [28]. However, in earlier works by Smith et al. they found out that *E. coli* accounts for 42% of UTI cases [29]. *Proteus* sp., with 36.4% prevalence, has a significant association with UTI. Its active motility and swarming ability can, in comparison with other organisms, traverse easily through the urethra. 

Results from the study revealed that the susceptibility of the isolates to the twelve antibiotics differs with the species. Higher proportions (95%) of bacterial isolate were sensitive to levofloxacin. It has been reported that levofloxacin shows excellent activity against the urinary pathogens [30]. Drugs like tetracycline, ampicillin, and co-trimoxazole show relatively fair activity on isolates with 4.96%, 4.96%, and 6.6% activity, respectively, although the recorded high level of resistance could be due to the cheap price and affordability of these drugs. These drugs are very common and accessible to the youth (and the general populace). The implication of this is the possibility of easy access causing self-medication, misuse, and abuse, leading to the development of resistance [31].

Although the findings of this study are comparable to previous reports, there were some limitations. This was a cross-sectional study conducted with a small sample size, which limited our ability to explain the causal correlations between risk factors and ASB. All participants came from one primary care center and thus, the findings may not represent all of Ghana.

## 5. Conclusions

Asymptomatic bacteriuria is prevalent among women of reproductive age and especially pregnant women in Akuapem-Mampong Municipality. *E. coli* and *Proteus* spp. were common ASB-causing uropathogens. It is therefore necessary to increase awareness through public health education at antenatal clinics and institutions on personal hygiene, cleanliness, as well as early and regular screening for uropathogens. Moreover, future studies are required to assess the impact on public health of the rate of bacterial infections.

## Figures and Tables

**Table 1:** Distribution of the presence of asymptomatic bacteriuria (ASB) stratified by socio-demographic characteristics.

Socio-Demographic Characteristics	Total (*n* = 300)	Women with ASB (*n* = 121)	Women with no ASB (*n* = 179)	*p*-Value	PRR (95% CI)	*p*-Value
Age (years) (Mean ± SD)	25.43 ± 6.54	24.20 ± 3.14	25.79 ± 2.01	<0.001		
Age groups (years)				<0.001		
<20	32 (10.7%)	7 (5.8%)	25 (14.0%)		1	
20–24	187 (62.3%)	97 (80.2%)	90 (50.3%)		2.4 (1.6−9.3)	0.002
25–29	35 (11.7%)	15 (12.4%)	20 (11.6%)		2.0 (0.9−7.8)	0.080
30–34	29 (9.7%)	0 (0.0%)	29 (18.4%)		0.1 (0.0−1.1)	0.011
35–39	17 (5.3%)	5 (1.6%)	12 (6.7%)		1.3 (0.4−5.7)	0.729
Highest Education level				<0.001		
None	17 (5.7%)	3 (2.5%)	14 (7.8%)		1	
Primary	47 (15.7%)	10 (8.3%)	37 (20.7%)		1.3 (0.3−5.6)	1.000
JHS	119 (39.5%)	63 (52.1%)	56 (31.3%)		3.2 (1.5−19.5)	0.006
SHS	81 (27.1%)	21 (17.4%)	60 (33.5%)		1.6 (0.5−6.7)	0.549
Tertiary	36 (11.9%)	24 (19.7%)	12 (6.7%)		4.0 (2.4−41.4)	0.001
Occupational status				0.061		
Unemployed	93 (31.0%)	33 (27.3%)	60 (33.5%)		1	
Informal employee	153 (51.0%)	81 (66.9%)	72 (40.2%)		1.5 (1.2−3.5)	0.009
Formal employee	54 (18.0%)	7 (5.8%)	47 (26.3%)		0.2 (0.1−0.8)	0.010
Residency				0.001		
Urban	23 (7.6%)	4 (3.3%)	19 (10.6%)		0.5 (0.1−1.4)	0.212
Rural	107 (35.7%)	34 (28.0%)	73 (40.8%)		1	
Urban Slum	170 (56.7%)	83 (68.7%)	87 (48.6%)		1.5 (1.2−3.4)	0.006
Marital Status				0.005		
Single	50 (16.7%)	14 (11.6%)	36 (20.1%)		1	
Married/Cohabited	242 (80.5%)	101 (83.5%)	141 (78.8%)		1.5 (0.9−3.6)	0.081
Divorced/Widowed/Separated	8 (2.8%)	6 (4.9%)	2 (1.1%)		2.7 (1.4−42.9)	0.016

JHS = Junior High School, SHS = Senior High School, PRR = Prevalence rate ratio, CI-Confidence level, *p*-value < 0.05 = statistically significant. 1: reference category.

**Table 2:** Distribution of ASB stratified by lifestyle characteristics and other factors.

Risk Factors	Women with ASB (*n* = 121)	Women with no ASB (*n* = 179)	*p*-Value	PRR (95%CI)	*p*-Value
Department attended			<0.001		
Antenatal	100 (82.4%)	114 (63.7 %)		1.9 (1.6−4.8)	<0.001
OPD	21 (17.6%)	65 (36.3%)		1	
Sexual relationship					
Yes	78 (64.5%)	163 (91.1%)	0.001	0.4 (0.1−0.5)	<0.001
No	43 (35.5%)	15 (8.9%)		1	
On birth control pills			0.240		
Yes	54 (44.6%)	93 (52.0%)		0.9 (0.5−1.2)	0.240
No	67 (55.4%)	86 (48.0%)		1	
Wash genital area after sex			<0.001		
Always	23 (19.0%)	97 (54.2%)		0.3 (0.1−0.4)	<0.001
Often	35 (28.9%)	48 (26.8%)		0.6 (0.2−0.8)	0.003
Once a while	63 (52.1%)	34 (19.0%)		1	
Place of defecation			0.208		
Public toilet	44 (36.4%)	52 (29.1%)		1.2 (0.9−2.3)	0.208
Private toilet	77 (63.6%)	127 (70.9%)		1	
Awareness on UTI			0.712		
Yes	81 (66.9%)	116 (64.8%)		1.1 (0.7−1.8)	0.712
No	40 (33.1%)	63 (35.2%)		1	
HM with UTI			0.684		
Yes	12 (9.9%)	15 (8.3%)		1.1 (0.5−2.7)	0.684
No	109 (90.1%)	164 (91.7%)		1	

*p*-value < 0.05 = statistically significant, PRR = Prevalence rate ratio, CI = Confidence interval, HM = House mate, UTI = Urinary tract infection, OPD = Out-patient department. 1: reference category.

**Table 3:** Urine dipstick findings for study participants.

Urine Dipstick Findings	Frequency (*n* = 300)	Percentage (%)
Protein	25	8.3
Blood	5	1.7
Glucose	7	2.3
Nitrite	90	30.0
Leukocyte	105	35.0
Ketone	5	1.7

**Table 4:** Distribution of anti-microbial activity against isolates obtained from study participants.

Antibiotics	*E. coli* (*n* = 57)	*Proteus* spp. (*n* = 44)	*Enterococcus feacalis* (*n* = 10)	*Klebsiella* spp. (*n* = 10)	Total Resistance Percentage (%)	Total Sensitivity (%)
Ampiciline	56 (98.25)	41 (93.18)	8 (80.00)	10 (100)	95.04	4.96
Co-trimoxazole	55 (96.49)	40 (90.91)	8 (80.00)	10 (100)	93.38	6.62
Tetracycline	54 (94.73)	41 (93.18)	10 (100)	10 (100)	95.04	4.96
Cefotaxine	3 (5.26)	2 (4.55)	0 (0.00)	2 (20.00)	5.79	94.21
Ceftixozime	3 (5.26)	2 (4.55)	0 (0.00)	2 (20.00)	5.79	94.21
Ciprofloxacin	5 (8.77)	6 (13.64)	1 (1.00)	3 (30.00)	12.39	87.61
Levofloxacin	2 (3.50)	2 (4.55)	0 (0.00)	2(20.00)	5.65	94.35
Nalidixic acid	4 (7.02)	1 (2.27)	2 (20.00)	2 (20.00)	8.13	91.87
Gentamicin	3 (5.26)	7 (15.91)	2 (20.00)	2 (20.00)	11.57	88.43
Amikacin	0 (0.00)	0 (0.00)	0 (0.00)	0 (0.00)	0.00	0.0
Cefuroxime	10 (17.54)	16 (36.36)	2 (20.00)	3 (30.00)	25.62	74.8
Nitrofurantoin	1 (1.82)	2 (4.55)	1 (10)	1 (10)	4.13	93.87

*n* = frequency, spp. = species.
